# The impact of information about different absolute benefits and harms on intention to participate in colorectal cancer screening: A think-aloud study and online randomised experiment

**DOI:** 10.1371/journal.pone.0246991

**Published:** 2021-02-16

**Authors:** Juliet A. Usher-Smith, Katie M. Mills, Christiane Riedinger, Catherine L. Saunders, Lise M. Helsingen, Lyubov Lytvyn, Maaike Buskermolen, Iris Lansdorp-Vogelaar, Michael Bretthauer, Gordon Guyatt, Simon J. Griffin

**Affiliations:** 1 The Primary Care Unit, Department of Public Health and Primary Care, University of Cambridge, Cambridge, United Kingdom; 2 Clinical Effectiveness Research Group, Department of Transplantation Medicine, Oslo University Hospital, and Institute of Health and Society, University of Oslo, Oslo, Norway; 3 Department of Health Research Methods, Evidence, and Impact, McMaster University, Hamilton, Canada; 4 Department of Public Health, Erasmus MC University Medical Center Rotterdam, Rotterdam, The Netherlands; Centro per lo Studio e la Prevenzione Oncologica, ITALY

## Abstract

**Background:**

There is considerable heterogeneity in individuals’ risk of disease and thus the absolute benefits and harms of population-wide screening programmes. Using colorectal cancer (CRC) screening as an exemplar, we explored how people make decisions about screening when presented with information about absolute benefits and harms, and how those preferences vary with baseline risk, between screening tests and between individuals.

**Method:**

We conducted two linked studies with members of the public: a think-aloud study exploring decision making in-depth and an online randomised experiment quantifying preferences. In both, participants completed a web-based survey including information about three screening tests (colonoscopy, sigmoidoscopy, and faecal immunochemical testing) and then up to nine scenarios comparing screening to no screening for three levels of baseline risk (1%, 3% and 5% over 15 years) and the three screening tests. Participants reported, after each scenario, whether they would opt for screening (yes/no).

**Results:**

Of the 20 participants in the think-aloud study 13 did not consider absolute benefits or harms when making decisions concerning CRC screening. In the online experiment (n = 978), 60% expressed intention to attend at 1% risk of CRC, 70% at 3% and 77% at 5%, with no differences between screening tests. At an individual level, 535 (54.7%) would attend at all three risk levels and 178 (18.2%) at none. The 27% whose intention varied by baseline risk were more likely to be younger, without a family history of CRC, and without a prior history of screening.

**Conclusions:**

Most people in our population were not influenced by the range of absolute benefits and harms associated with CRC screening presented. For an appreciable minority, however, magnitude of benefit was important.

## Introduction

Many countries have introduced population wide screening programmes for a number of cancers, including colorectal (CRC) [1], breast [2], and cervical [3] cancers. These reduce disease specific mortality and, in the case of CRC and cervical cancer, disease specific incidence, at a population level [4–7]. However, within the general population, individuals’ risk of developing CRC varies considerably depending on factors such as age, sex, body mass index (BMI), lifestyle and genetics [8]. The potential benefits of screening therefore vary substantially between individuals. All screening programmes are also associated with harms. These include direct harms to those screened as well as indirect harms through diversion of resources away from other services. Direct harms to those screened include complications arising from the screening tests and/or subsequent investigations, the identification or treatment of conditions that may never cause illness (overdiagnosis and overtreatment) and psychological consequences. These harms also vary with age and the presence or absence of co-morbidities. There are, therefore, potentially large differences between individuals in the potential absolute benefits and harms of screening, and thus in the net benefit.

This individual level variation in the absolute benefits and harms of screening has led to two proposals. The first is for a shift in screening recommendations from promoting uptake by emphasising average population level benefits towards enabling an approach in which individuals receive support to make informed decisions based on personalised estimates of benefits and harms [9, 10]. The second is risk-stratified screening, in which the age of first invitation, the choice of test and/or the screening interval are tailored to an individual’s risk [11].

By design, supporting more individualised informed decision making requires individuals to be informed of the absolute benefits and harms from screening. Introducing risk-stratified screening, in particular using risk to determine the age at first invitation, would also likely require communication of risk. Although providing information on risk may influence the decision to take up screening at an individual level and support shared decision-making [12, 13], many in the general population do not easily understand the concept of risk or probability [14, 15] and even individuals who appear to understand and recall risk information often do not believe that the information reflects their own risk [16]. Understanding how individuals use information about benefits and harms of screening and how the magnitude of the absolute benefits and harms influences uptake is therefore important.

Existing research in this area has largely focused on stated choice studies (most commonly discrete choice analyses [17–20]). These studies have shown that the type of screening test, preparation required, screening interval, and risk reduction all influence screening preferences, with the risk reduction the most important attribute in one study [17]. There are, however, a number of limitations with these studies. In particular, participants were rarely presented with absolute estimates of effect or potential harms, nor with graphical representations of risk. Furthermore, the risk reductions were presented as variations in relative-risk reduction from a fixed population average absolute risk, rather than variations in absolute risk with a fixed relative-risk reduction. Relative risk formats have consistently been shown to produce more favourable evaluations of treatment options than absolute risk estimates [21] and graphical representations to increase accuracy when making treatment decisions [22]. Moreover, in practice the relative-risk reduction for a given screening test is similar across risk groups while the absolute risk varies. The findings from these existing studies, therefore, provide limited information to inform screening programmes.

To address these gaps in the literature, we used CRC screening as an exemplar to explore how people make decisions about screening following presentation of information about different levels of absolute benefits and harms. We also aimed to determine the extent to which preferences for screening vary with different levels of absolute risk of developing disease, between different screening tests and between individuals. We hypothesised that there would be considerable variation in how individuals use this information to make decisions regarding CRC screening and the relative weight they attribute to the benefits, harms and burdens of screening options. Further, we hypothesised that individuals would be more likely to opt for screening at higher absolute risks of developing CRC and that those who have attended screening in the past or have a family history of CRC will be more likely to opt for screening at lower absolute risks.

## Methods

We conducted two linked studies. The first was a think-aloud study in which participants were encouraged to verbalise their thought processes while completing a survey in which they were presented with scenarios presenting different absolute benefits and harms of CRC screening and asked to make decisions about screening. As well as providing in-depth data on how individuals use the information regarding benefits and harms to make decisions, that study also enabled us to pilot the survey and refine it prior to the second study, an online randomised experiment designed to quantify: 1)the extent to which intention to attend screening varies with levels of absolute risk and between screening tests (the primary outcome); and 2) the extent to which participant-level characteristics or the different screening tests were associated with different patterns of responses across the three risk levels (the secondary outcome).

### Part 1. Think-aloud interviews

#### Study design

Participants completed an online survey while “thinking-aloud” about their internal thought processes. This think-aloud method originated within the field of psychology [23] as a basis for investigating the mental processes underlying complex task performance. By asking participants to verbalise their thoughts and spontaneously report what goes through their minds while performing a task, the approach provides rich data on cognitive processes. It has been widely used in many scientific disciplines, including how respondents make benefit-risk trade-offs in discrete choice experiments [24]. It is, therefore, an appropriate method to explore in-depth how people make decisions about screening.

#### Participants and recruitment

Through a market recruitment company (iPoint Market Research, www.i-point.co.uk), we recruited 20 members of the public between 45–79 years of age without a history of inflammatory bowel disease, hereditary non-polyposis colorectal cancer or familial adenomatous polyposis. Participants were purposively sampled by age, gender, ethnicity, education background and prior screening history.

#### Survey

We developed the survey used for the think-aloud study in collaboration with our patient or public involvement (PPI) members. S1 File provides full details of all the questions and measures. The survey began with a series of questions on key personal characteristics: age, sex, ethnicity, education level, family history of CRC, numeracy (Schwartz scale [25]), perception of their own CRC risk (assessed on a 7-point Likert scale from likely to unlikely), cancer worry (Lerman cancer worry scale [26]), and whether they had attended and/or been invited for CRC screening. Validated instruments and questions were used for these questions where possible.

Participants then reported what they knew about CRC screening before reviewing details regarding CRC. To ensure that participants were able to understand what each test involves and make an informed decision about whether to take up screening, this included a description of the three most common screening tests (colonoscopy, sigmoidoscopy and Faecal Immunochemical Testing (FIT)) and their associated burdens/harms. Online information related to the Bowel Cancer Screening Programme in England reviewed by our PPI members informed the descriptions of the screening tests. A literature review informed the burdens and harms (Web Appendix 5 in [10]). Burdens included preparation prior to each test, what is involved during the test, how long having the test would take, any likely pain and any need to rest afterwards. Harms for FIT included those resulting from colonoscopy if a FIT test came back positive and for both sigmoidoscopy and colonoscopy included the risk of bleeding or bowel perforation. S1 File provides full details. After reading that information, participants provided a global rating of the burden, inconvenience or worry they associated with each screening test on a 5-point Likert scale from 1 “No inconvenience, burden or worry” to 5 “Very great inconvenience, burden or worry”.

Participants then saw nine scenarios that presented the absolute benefits and harms associated with three different 15-year absolute risks of developing CRC (1%, 3% and 5%) for each of the three screening tests (FIT, sigmoidoscopy and colonoscopy). The scenarios were grouped by screening test with participants randomised to groups with one of the following orders: 1) FIT, sigmoidoscopy, colonoscopy; 2) sigmoidoscopy, colonoscopy, FIT; or 3) colonoscopy, FIT, sigmoidoscopy. The order in which participants saw the three 15-year absolute risks of developing CRC within each of these groups was randomly allocated with each participant being randomised to one of the following orders: 1) 1%, 5%, 3%; 2) 5%, 3%, 1%; or 3) 3%, 1%, 5%. The order in which participants saw the 15-year absolute risks of developing CRC was the same for each individual for all of the three screening tests. The range 1–5% 15 year risk was chosen because this covers the range of risk levels observed in the general population [27] and we wanted to assess the potential impact of providing accurate risk information within screening programmes.

**Fig 1:**
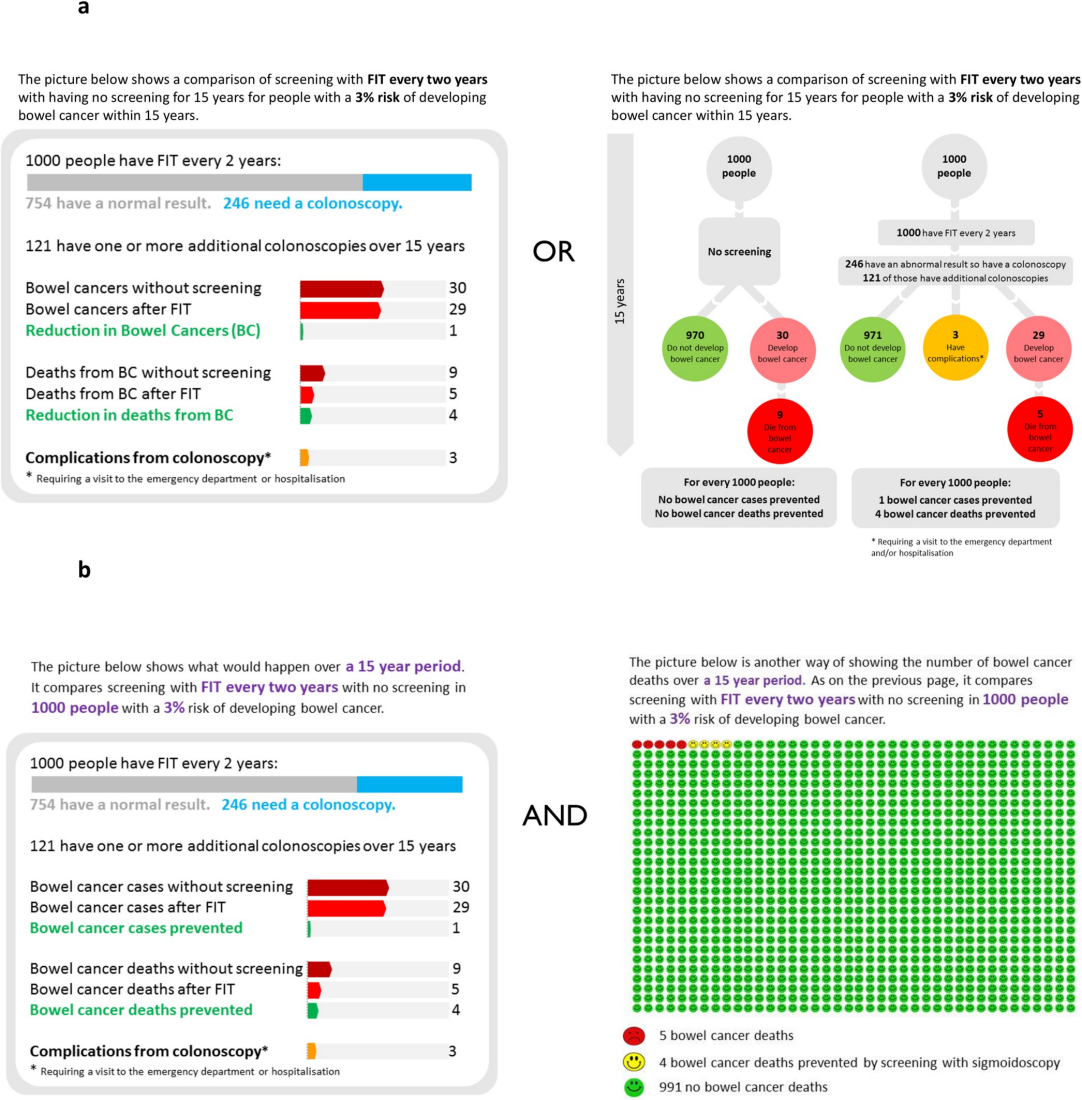
Formats of risk presentation. Examples of the formats of presentation of absolute benefits and harms for faecal immunochemical testing (FIT) and 3% baseline absolute risk used in a) The Think-aloud study and b) The online survey experiment.

Each scenario was presented in a graphical representation that included estimates of the absolute risk of CRC incidence and mortality, the risk reduction achieved through screening, and the risk of complications requiring a visit to the emergency department or hospitalisation. In order to inform the design of the graphical representation for the subsequent online experiment, we developed two graphical representations for the think-aloud study, a bubble format and a bar format (Fig 1A), both informed by the literature addressing risk presentation [28–31]. The bubble format was based on the information provided in the English Breast Cancer Screening participant information leaflet that was developed during a four-step process involving experts and members of the public [32]. The bar format was included as an alternative to enable us to explore whether a more visual comparison of the absolute benefits and harms influenced how people understood and used the information. Prior to their use in the study, both formats were piloted with our PPI representatives. They suggested a number of modifications, including clarifying that complications were those requiring a visit to the emergency department or hospitalisation and including details of the number of people with a normal FIT test. These modifications were incorporated prior to data collection.

Participants were randomly assigned to one of the two formats, seeing all nine scenarios in that format. In both formats, published data from a microsimulation study based on the Microsimulation Screening Analysis-Colon model (MISCAN-Colon) [33] informed estimates for the absolute benefits and harms (S1 Table).

After viewing each of the nine scenarios, participants were asked whether, on the basis of the information, they would undergo screening “*Based on this information*, *would you choose to go for screening*? *Yes/No*”.

To test understanding of the risk information, participants completed a test question after the first scenario in which they were asked to select the correct number of CRC cases that would be prevented in the scenario from four options. We additionally included an attention check (“*It is important that you pay attention in this study*. *Please tick ‘strongly disagree’*”) early in the questionnaire to identify inattentive participants [34].

#### Data collection

Using a discussion script as a guide (S2 File), an experienced qualitative researcher (KM) conducted face-to-face interviews in participants’ homes between 25^th^ April and 8^th^ May 2019. The interviewer initially confirmed participation and consent and then asked a warm up question (“What did you to today before coming to the session?”) to build rapport. Participants then completed the survey online on a tablet computer in front of the researcher while she prompted them to verbalize their thoughts through encouraging questions such as “What are you thinking when you are looking at this image?” and “Can you describe your reasons for choosing this answer?”. The interviews were audio-recorded using encrypted audio-recorders and then transcribed verbatim.

#### Analysis

The transcripts were analysed using Thematic analysis [35]. One researcher (KM) read the transcripts from early interviews to identify themes and developed an initial coding frame. Aided by NVivo software [36], KM then coded the remainder of the transcripts using that coding frame. This was followed by an interpretation stage during which she read through the coded categories and identified the main concepts and ways in which different parts of the data were related to each other. Three members of the research team (LL, LH and MB) read a selection of transcripts and the four team members, and then the wider team, discussed the identified themes.

### Part 2. Online survey

#### Study design

This was a randomised parallel group online experiment in which participants completed a shorter version of the survey used for the think-aloud study that included only three scenarios covering three different 15-year absolute risks of CRC, all relating to the same screening test and all with the bar presentation format alongside a pictograph (Fig 1B). The team made the decision to use the bar presentation format alongside a pictograph and this shortened version in which participants only saw three scenarios following analysis of the data from the think-aloud study in part 1 and based on concern that in an online experiment participants would lose interest if presented with all nine scenarios.

**Fig 2:**
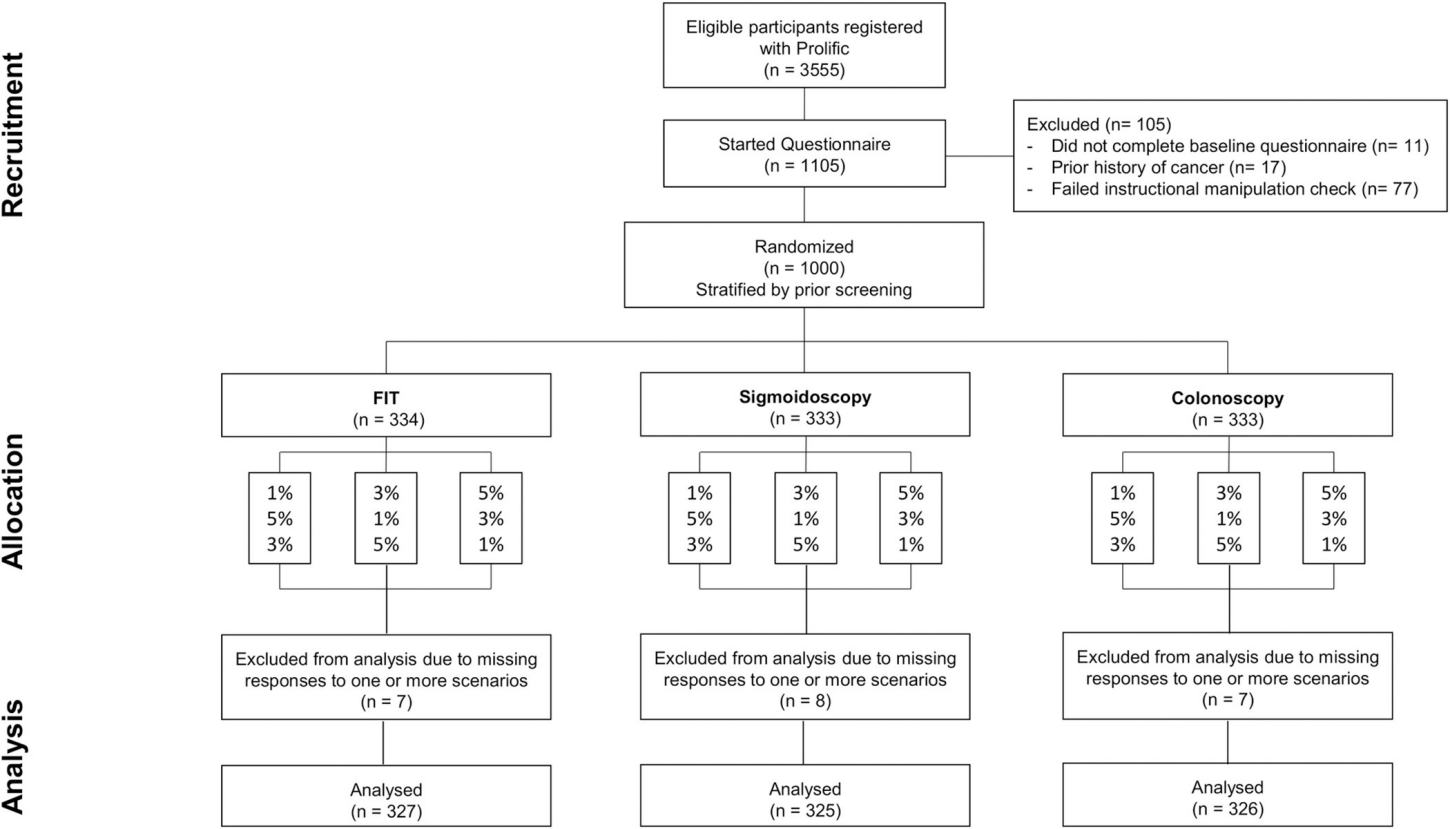
Participant flow diagram for online survey experiment.

Stratified by whether they had been invited to screening in the past, participants were randomised 1:1:1 at an individual level based on computer generated random numbers in block sizes of three to one of FIT, sigmoidoscopy or colonoscopy scenarios. The order in which participants viewed the scenarios was also randomly allocated using the same method according to the absolute level of risk, with participants either viewing 1% followed by 5% then 3% (1–5–3), 3% followed by 1% then 5% (3–1–5) or 5% followed by 3% then 1% (5–3–1) (Fig 2).

#### Participants and recruitment

We recruited 1000 participants who had not taken part in the think-aloud study through an online participant recruitment platform developed for research surveys and market research (www.prolific.ac) between 7^th^-8^th^ June 2019. Participants were eligible to take part if they were between 45–79 years of age; resident in the UK without a past history of cancer; and had a Prolific approval rating ≥95%. The Prolific approval rating is a measure of the proportion of studies completed by the participant that have subsequently been approved by the study team. Exclusion of participants with a low approval rating is considered good practice [37].

#### Data collection

As for the think-aloud study, the questions and scenarios were embedded within an online survey. Except for the reduced number of scenarios, the questions were the same as for the think-aloud study (S1 File). Participants who failed to answer the attention check correctly were excluded prior to randomisation.

#### Analysis

Using the three responses from all participants, we first described the overall proportion of participants expressing intention to attend screening at each risk level. To enable us to explore the primary outcome, the extent to which intention differed with different levels of absolute risk of developing CRC and between screening tests, we used multivariable logistic regression with intention as the outcome and the risk level and screening test as explanatory variables, accounting for clustering at participant level using cluster robust standard errors and adjusting for whether participants had previously been invited for CRC screening (the stratification variable used for randomisation) and the order in which participants were presented with the different risk levels. Hypothesizing that the order in which the risk levels were presented might influence the response to a given risk level (for example, that intention to attend at a risk level of 3% would be different when that was the first scenario presented versus if it followed a risk level of 5%), we also tested for an interaction between the order and the risk levels. Where significant, we present the results stratified by the order in which the risk levels were presented.

To explore patterns in responses at an individual level, we then grouped participants into: 1) those who intended to take up screening at all three risk levels; 2) those who did not intend to take up screening at any risk level; and 3) those whose responses varied with risk level. To identify whether patient-level characteristics or the different screening tests were associated with different patterns of responses across the three risk levels (secondary outcome) we used the Chi-squared test (unadjusted) and multinomial logistic regression (adjusted) to test for associations between the likelihood of being in each of the three groups of response patterns and the screening test (FIT, sigmoidoscopy or colonoscopy) and individual level characteristics (age, sex, ethnicity, university level education, family history of CRC, prior history of screening for CRC, numeracy, understanding of risk information and prior perceptions of screening).

In an exploratory analysis, we repeated all analyses stratified by whether participants correctly answered the test question. We report all regression analysis results as odds ratios (ORs) with 95% confidence intervals or p values. All analyses were performed using Stata Version 14 [38] with statistical significance set at p<0.05.

#### Sample size

When designing the study, assuming an overall uptake of screening of 66% [39] and intention to attend screening of 80%, 83% and 86% in groups with the three levels of risk respectively, we estimated that 1000 participants would give us 90% power to detect a difference in intention across the different levels of risk.

### Ethical approval and consent

The Cambridge Psychology Research Ethics Committee approved the study (PRE.2019.022). All participants provided written online consent at the start of the study.

## Results

### Think-aloud study

The mean age of the 20 participants was 61.7 years (range 45–77 years). Eleven were female and 17 were white British and had completed either secondary (n = 9) or university level (n = 7) education. Ten had attended bowel cancer screening in the past. Fifteen correctly answered the test question.

Despite verbalising the numbers presented in the scenarios, for many the information on absolute benefits and harms did not appear to be a factor in their decision making. Twelve intended to take up screening either again or for the first time in the future with all three screening tests at any level of risk.

“*I’ve already said that nothing would stop me going for a screening*. *So the statistics are really irrelevant*. *Whether it’s 1 in 1*,*000 or 1 in a million*, *if there’s a chance of preventing it then I would take no risk*.” (Male, 60–64, prior CRC screening)

In most cases these participants appeared to be making decisions based on prior beliefs around screening in general and did not appear to consider the specific burden or harms of screening. Even when they mentioned the harms of the tests when explaining their decision, they dismissed these almost immediately and described instead how they believed the benefits outweighed any risks.

“*The only thing that’s influencing me is my thoughts on screening and I still think the benefits of being screened outweighs the complications*.” (Female, 65–69, no prior CRC screening)

In these cases the benefits of screening were often described in the context of family members’ experiences of the tests and personal concerns for developing cancer in the future. Again, the absolute magnitude of these benefits did not appear to matter. Instead, a common view was that as long as one person benefitted it was worth it because they could be that person.

“*It could be me*. *That death could be me*. *It could be my family member*. *So*, *even if it prevents one death*, *I’d still do it*.” (Male, 60–64, prior CRC screening)

For some of the participants (n = 7), the absolute benefits and harms and screening tests did appear to influence their decisions.

“*Well*, *if that [1%] was the percentage I’d think*, *“Well*, *is it really worth it*?*” The one doesn’t increase your chance one way or the other*, *really*, *for having it*, *or not being screened*.” (Male, 65–69, no prior CRC screening)

“*Maybe I’d say a no for three*, *but if I was a five per cent*, *I might think about it*.” (Female, 45–49, no prior CRC screening)

In many of these cases, decisions were not based on the risk level alone. Instead participants were influenced by their previous experiences of the tests and the test characteristics. For example, one participant felt that they would only accept screening with a colonoscopy at 5% risk and would decline screening with FIT or sigmoidoscopy at that level because of a fear of false-positives. Another appeared to substitute their own beliefs for the evidence provided, not accepting screening with FIT below 5% risk because they felt it was not effective at lower risk levels. One participant did not wish to attend screening for any of the tests at any risk level for fear of complications from sigmoidoscopy and colonoscopy and a previous false positive from a FIT test.

There were no clear patterns in the data with participant level characteristics or between the two graphical representations. Some participants, however, found it difficult to follow the bubble format. In particular, they did not appear to appreciate that some of the people who had an abnormal result would not develop cancer.

### Online experiment

At the time of recruitment, 3,555 participants met the eligibility criteria for the study. Of these, 1105 started the questionnaire and 1000 were randomised. 22 participants did not complete responses for all three scenarios. 978 participants are therefore included in the analysis (Fig 2). The characteristics of the 978 participants were balanced across each group, with no substantial difference from the 22 who did not complete all three scenarios (S2 Table). Most were aged between 45–65 years, female, of white ethnicity and without a family history of CRC. Half had university level education. 61% (600/977) thought the benefits of CRC screening outweighed potential harms for everyone. 51% (502/978) selected the correct response to the test question. In univariable analysis, participants with high numeracy were more likely to select the correct response than those with low numeracy (OR 1.36 (95% CI 1.00–1.85). There was no association with age group, sex, ethnicity or university level education and the association with numeracy failed to reach statistical significance in multivariable analysis including age, sex, ethnicity and university level (AOR 1.39 (95% CI 0.98–1.89)).

#### Response to scenarios

**Fig 3:**
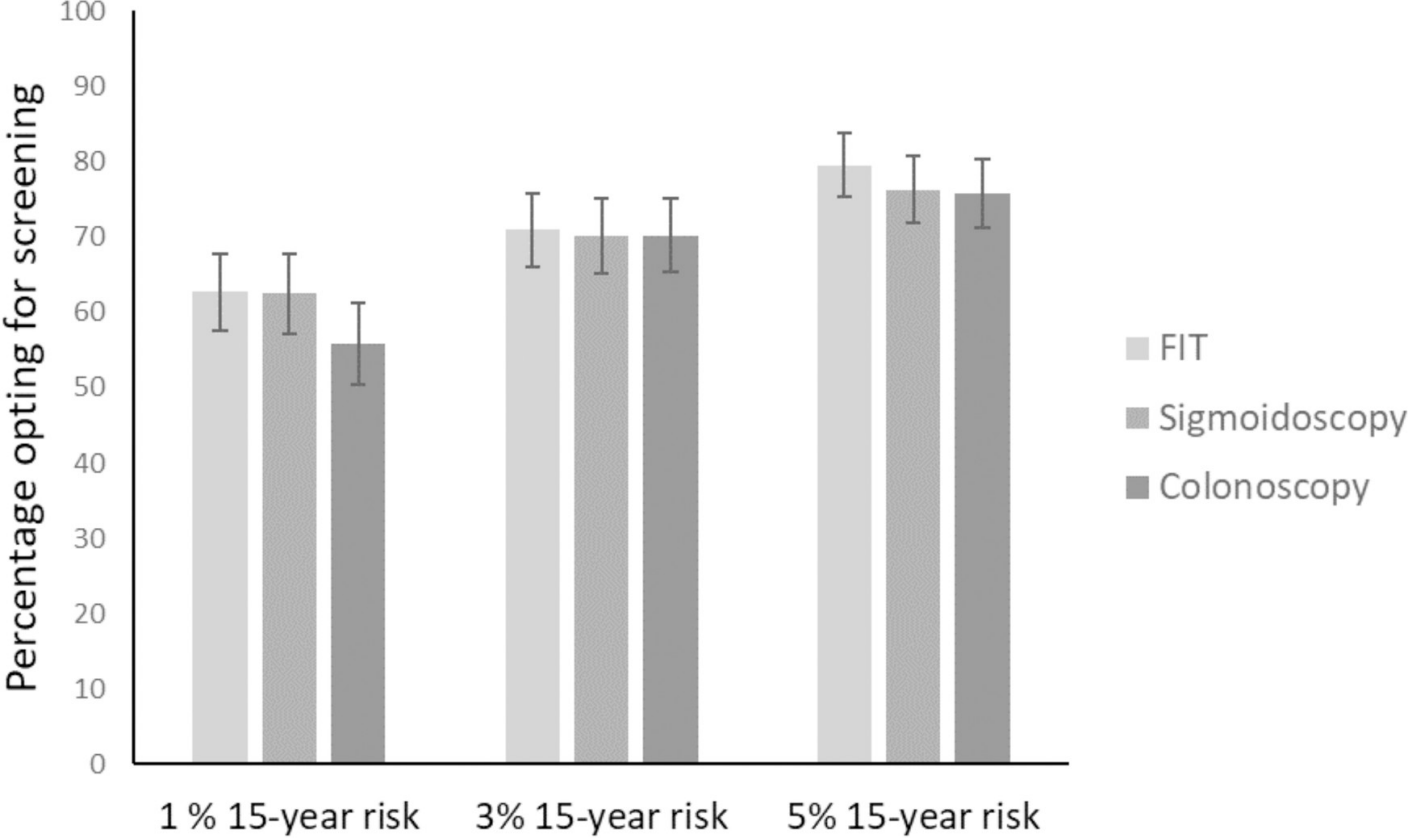
Percentage (± 95% confidence interval) of participants expressing intention to attend screening for each baseline risk level and screening test.

The number of participants expressing intention to take up screening in each of the nine study groups for all three scenarios is shown in S3 Table. Including the three responses from all participants across all three screening tests, 60% (95% CI 57.2–63.4%) expressed intention to attend screening at 1% 15-year risk, 70% (95% CI 68.4–74.2%) at 3% and 77% (95% CI 74.4–79.8%) at 5% (Fig 3). In multivariable analysis there were no significant differences in intention between the three screening tests (OR 0.93 ((95% CI 0.70–1.25) for sigmoidoscopy compared with FIT and OR 0.83 (95% CI 0.62–1.11) for colonoscopy compared with FIT) but both the baseline risk level and the order in which participants were presented with the different risk levels were significantly associated with intention to attend screening. A significant interaction existed between the risk level and order in which the participants were presented with the different risk levels. Stratifying the results by the order in which the risk levels were presented showed that in all three orders participants were more likely to take up screening as the risk level increased (Tables 1 and S4). The relative difference in intention between the three risk levels varied with the order, with the strongest association between risk and intention seen in the groups who saw 5% followed by 3% followed by 1%.

**Table 1:** Odds ratios (ORs) of intention to attend screening at each baseline 15 year percentage risk of CRC and for each order in which participants were presented with the three scenarios.

15-year risk (%)	Order in which participants were presented with the risk levels		
	1—5—3	3—1—5	5—3—1
1	1 (ref)	1 (ref)	1 (ref)
3	1.17 (0.95 to 1.44)	1.75 (1.45 to 2.10)	1.83 (1.49 to 2.25)
5	1.56 (1.22 to 2.00)	2.31 (1.83 to 2.92)	2.92 (2.28 to 3.74)

ORs are adjusted for screening test, previous invitation to screening and stratified by baseline percentage risk and order.

Repeating this analysis stratified by response to the test question after the first scenario showed that the association between risk level and intention to take up screening was slightly stronger in those selecting the correct response (S5 Table).

**Fig 4:**
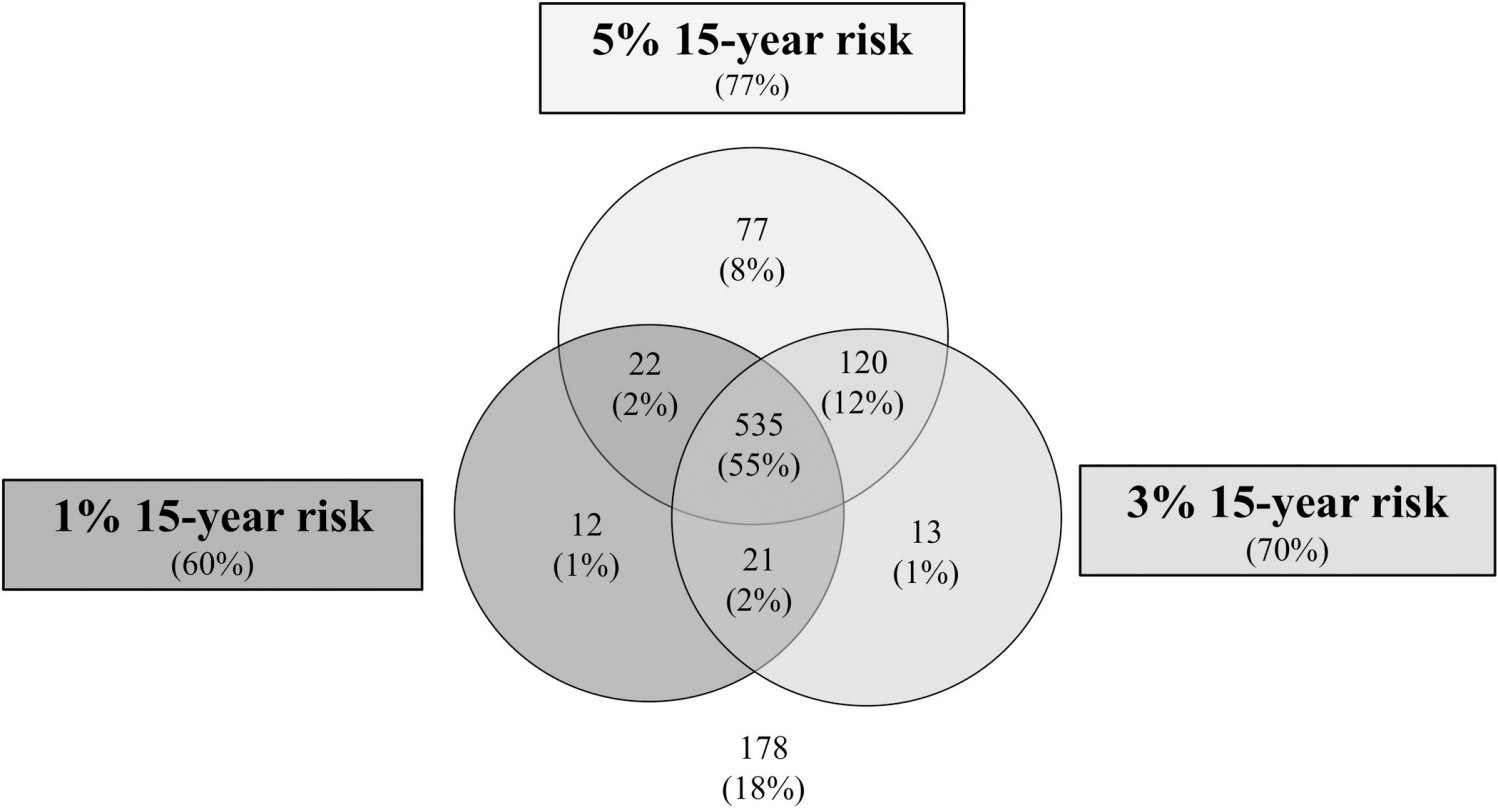
Participants expressing an intention to attend screening at each risk level across the three scenarios for FIT, sigmoidoscopy and colonoscopy combined.

Grouping the participants who saw scenarios relating to FIT, sigmoidoscopy and colonoscopy, Fig 4 shows the patterns of responses across the three risk levels for each individual participant. 535 (54.7% (95% CI 51.5–57.9%)) individuals said yes to all three scenarios and 178 (18.2% (95% CI 15.8–20.8%)) said no to all three scenarios. 265 (27.1% (95% CI 24.3–30.0%)) individuals responded differently across the three scenarios, suggesting that the absolute benefits and harms influenced their decision. Among those, 120 said no at 1% but yes at 3% and 5% (indicating a threshold between 1–3%) and 77 said no to 1% and 3% but yes to 5% (indicating a threshold between 3–5%). 68 provided inconsistent responses saying yes at either 1% or 3% and no at 3% or 5%.

Table 2 shows the characteristics of the participants in each of those response groups (always yes, always no and scenario dependent). In unadjusted analysis, the distribution of participants within those three groups varied with screening test, ethnicity, family history of CRC, prior history of screening, numeracy and prior beliefs around screening. These observed differences with ethnicity, family history of CRC, prior history of screening and prior beliefs about screening persisted in multivariable analysis (Tables 2 and S6). Participants with a family history of CRC and those who had attended screening previously were more likely to say yes at all risk levels and those of non-White ethnicity were more likely to respond differently across the three risk levels. Additionally, age was significant in multivariable analysis, with older individuals more likely to say no to all scenarios. There was no association between response group and whether or not participants answered the test question correctly.

**Table 2:** Response patterns to the scenarios stratified by study variables.

Variable	Always no (*N* = 178) *n* (unadjusted %)	Scenario dependent (*N* = 265) *n* (unadjusted %)	Always yes (*N* = 537) *n* (unadjusted %)	*p* (unadjusted analysis)	*p* (adjusted analysis*)
Screening test				**0.044**	0.085
FIT	48 (14.7)	94 (28.8)	185 (56.6)		
Sigmoidoscopy	67 (20.6)	72 (22.2)	186 (57.2)		
Colonoscopy	63 (19.3)	99 (30.4)	164 (50.3)		
Age (years)				0.294	**0.002**
45–54	95 (16.6)	166 (29.0)	311 (54.4)		
55–64	61 (19.4)	79 (25.2)	174 (55.4)		
>65	22 (23.9)	20 (21.7)	50 (54.4)		
Sex				0.085	0.063
Female	125 (19.4)	183 (28.4)	336 (52.2)		
Male	53 (15.9)	82 (24.6)	199 (59.6)		
Ethnicity				**0.028**	**0.046**
White	172 (18.4)	246 (26.3)	517 (55.3)		
Non-white	6 (16.2)	17 (46.0)	14 (37.8)		
University level education				0.454	0.92
Yes	89 (17.6)	146 (28.8)	272 (53.7)		
No	89 (18.9)	119 (25.3)	263 (55.8)		
Family history				**0.001**	**0.013**
No	163 (18.7)	247 (28.3)	462 (53.0)		
Yes	12 (14.0)	11 (12.8)	63 (73.3)		
Prior history of screening				**<0.0001**	**0.0002**
No	159 (20.9)	217 (28.5)	385 (50.6)		
Yes	19 (8.8)	48 (22.1)	150 (69.1)		
Numeracy				**0.034**	0.051
Low	45 (21.7)	42 (20.3)	120 (58.0)		
High	133 (17.3)	222 (28.9)	413 (53.8)		
Understanding of information				0.723	0.58
Incorrect	90 (18.9)	124 (26.1)	262 (55.0)		
Correct	88 (17.5)	141 (28.1)	273 (54.4)		
Prior perceptions of screening				**<0.0001**	**<0.0001**
Yes for all	44 (7.3)	160 (26.7)	396 (66.0)		
No for all	4 (20.0)	4 (20.0)	12 (60.0)		
It depends	130 (36.4)	101 (28.3)	126 (35.3)		

* Adjusted for all factors included in the table in addition to the order in which the scenarios were presented.

## Discussion

Consistent with our hypothesis, these two linked studies have shown that there is considerable variation in how individuals use information regarding absolute benefits and harms of screening to make CRC screening decisions. Overall individuals in the online experiment were more likely to intend to take up screening at higher absolute baseline 15-year risks of developing CRC. However, many (up to half of participants in both studies) did not fully understand the information and, within the range of absolute benefits and potential harms presented, the majority did not vary their decisions about whether to take up screening on the basis of the range of the number of cases or deaths from CRC that screening could prevent or the potential risk of complications from screening tests. Data from the think-aloud study suggests that for these individuals, their prior perceptions of screening and, for those opting for screening, the view that screening is worthwhile as long as one person might benefit, exert greater influence.

For a significant minority of people (27% in the online experiment), the absolute benefits and harms from screening do appear to be influential. Consistent with our hypotheses, these individuals are more likely to not have a family history of CRC and not have attended CRC screening in the past. Additionally, they are more likely to be younger and not believe that CRC screening is beneficial for everyone.

Notably, despite following best-practice [28, 31] and piloting the presentation formats with PPI members, many of the participants were unable to correctly select the number of cases of CRC prevented in the first scenario. Moreover, whether or not participants were influenced by the absolute benefits and harms was independent of the responses to that test question. The association between risk level and intention to take up screening was only slightly stronger in those who selected the correct response. That five of the 20 participants in the think-aloud study failed to provide a correct answer despite actively talking through the data suggests that these incorrect responses may reflect a lack of understanding rather than a lack of attention. Even among the group for whom the absolute benefits and harms appear to be influential, how the quantitative information influenced decision-making remains uncertain.

Although this is the first study to our knowledge to directly explore the impact of different absolute benefits and harms from screening among individuals, these findings are consistent with a previous online scenario-based study in which only 23% of 1,675 female participants varied their decisions on risk reduction mastectomy according to baseline risk of breast cancer mortality and the expected risk reduction due to mastectomy [40]. Surveys and focus groups in the context of breast cancer screening [41, 42], reviews of personalised risk communication [43, 44], and the wider decision-making literature [45–47] have also documented that decisions are often influenced by emotions and attitudes not by quantitative risk-based information… The difficulty lay individuals have understanding quantitative risk-based information is also well known [14], with the high proportion of participants who did not get the test question correct in this study is consistent with assessment of knowledge in a randomised trial of a web-based decision aid for breast cancer screening in which less than 30% of women correctly identified the number of women who will avoid dying from breast cancer because of screening or how many women not undergoing screening will die from breast cancer [48]. An important addition to the literature that this study provides is characterisation of those individuals for whom risk information does and does not appear to influence decisions concerning screening, enabling targeting of future interventions.

A key strength of this study is the mixed-methods design, incorporating both a qualitative think-aloud study to explore individuals’ thoughts and reasoning in depth and pilot the survey and a quantitative online experiment to describe patterns in responses and associations with participant level characteristics.

There are, however, a number of limitations. First, we only assessed intention within the range from 1% to 5% 15-year risk. While this range covers the average 15-year risk of CRC in UK adults between the ages of 50 and 74 years [27] and the think-aloud study findings suggest that the magnitude of the risk was not important to individuals whose responses did not vary with risk, we do not know if those individuals who appeared to be insensitive to risk may have thresholds outside this range. Secondly, although our recruitment strategy allowed for purposive sampling for the think-aloud study and a large sample size for the survey, the demographics of members of recruitment agencies are not necessarily representative of the UK population [49]. Additionally, over 90% of our population were of white ethnicity and few were over 60 years old. The large sample size also increases the chance of detection of small but unimportant effects. However, the magnitude of the differences we observed are substantial and comparable with the effect sizes seen in studies designed to increase uptake of screening [50, 51].

Thirdly, we conducted this study in the UK where there is an existing CRC screening programme. Most people (70%) were aware of this national programme and exposure to recommendations for screening may have influenced their views, particularly as there is less controversy surrounding CRC screening programmes than other screening programmes such as breast cancer. Fourthly, we considered only one timeframe of 15 years. Studies in cardiovascular disease have shown that shorter timeframes may result in more accurate risk perceptions and increased intention to change behaviour, especially for older people [52]. It is therefore possible that different timeframes would have different influences on individuals’ decision making, especially when considering different age groups. Finally, the study required participants to imagine that each scenario related to them and we measured intention rather than behaviour. Such hypothetical intention rates may not reflect subsequent uptake [53].

## Conclusion

The majority of people in these two studies were uninfluenced by the range of absolute benefits and harms associated with CRC screening presented. This suggests that providing additional information on absolute benefits and harms, either in the context of initiatives to improve informed choice or in risk-stratified approaches, may have little impact on the decisions the majority of people make about screening. These findings also raise important future research questions around the meaning and measurement of informed choice in the context of screening. Traditional measures of informed choice consider individuals to have made an informed choice when their knowledge, attitudes and behaviour are consistent [54]. Our findings suggest that individuals may have adequate knowledge and this may be consistent with their attitudes, but that they may not be using that knowledge to make their decision.

For an appreciable minority, however, the magnitude of benefit appeared to be important. These individuals are more likely to be younger, to not have a family history of CRC and to not have previously attended screening. Providing information on absolute benefits and harms when people are first invited to screening may therefore have a greater impact on decision making than providing it at subsequent invitations. The high proportion of participants, including those for whom the magnitude of benefit was important, who did not understand the information presented in this study, also highlights the need for further research into how best to present the information and the potential challenges of using such information to incorporate risk stratification into screening programmes.

## Supporting information

S1 File Online survey. (PDF)

S2 File Think Aloud study discussion script. (PDF)

S1 Table Estimates of absolute benefits and risks of screening presented within the scenarios. (PDF)

S2 Table Characteristics of participants for online survey. (PDF)

S3 Table Intention to attend screening within each study group across all three scenarios. (PDF)

S4 Table Adjusted percentages ± 95% CI* for participants intending to attend screening at each baseline 15 year percentage risk of CRC and for each order in which participants were presented with the three scenarios. (PDF)

S5 Table Odds ratios (ORs) of Intending to attend screening at each baseline percentage risk and for each order in which participants were presented with the three scenarios for participants who got the test question correct or incorrect. (PDF)

S6 Table Adjusted percentages for response patterns to the scenarios. (PDF)
