# Supplementary material for: The impact of information about different absolute benefits and harms on intention to participate in colorectal cancer screening: A think-aloud study and online randomised experiment
Source: PLoS One. 2021 Feb 16;16(2):e0246991. doi: 10.1371/journal.pone.0246991 (PMC7886213; doi:10.1371/journal.pone.0246991)
Supplement: S1 File — (PDF) [file pone.0246991.s001.pdf]

Online survey

[Note that this content was delivered online so the formatting looked different to in this document. The participants did not see the headings in grey boxes or the references but these are included here to illustrate where the questions have come from.]

Thank you for agreeing to complete this questionnaire. Please answer every question. If you are uncertain about how to answer a question, then please select the closest option.

ABOUT YOU AND YOUR HEALTH

The first part of the questionnaire asks for some background information about you so that we can include the views of a wide range of people in the study.

Have you ever had bowel cancer?

- Yes*
- No

*If the participant answers YES to this question they will see the following text:

Unfortunately you are not eligible to take part in this study. Thank you for your interest though and for reading this far.

Are you…

- Female
- Male

How old are you?

What is your ethnic group? Choose one option that best describes your ethnic group or background.

- White
- Mixed / Multiple ethnic group
- Asian / Asian British
- Black / African / Caribbean / Black British
- Other, please describe

What is your highest education level

- No formal education
- Primary education (educated to age 11 or before)
- Secondary education (educated to age 18 or before)
- University education

Have your parents or any brothers or sisters ever had bowel cancer?

- Yes
- No
- Don't know/prefer not to answer

Have you ever been told you have inflammatory bowel disease, hereditary non-polyposis colorectal cancer or familial adenomatous polyposis?

- Yes
- No

ABOUT YOUR VIEWS ON BOWEL CANCER AND SCREENING HISTORY

Bowel cancer is the term used for cancer that begins in the large bowel. It is also called colorectal cancer.

How likely do you think is it that you will get bowel cancer at some point in the next 10 years?

Likely |—|—|—|—|—|—| Unlikely

For each of the following statements, select the option that best applies to you: (Lerman cancer worry scale[1])

	Not at all	Rarely	Sometimes	Often	A lot
**During the past month, how often have you thought about your own chances of getting bowel cancer?**					
**During the past month, how often have thoughts about your chances of getting bowel cancer affected your mood?**					
**During the past month, how often have thoughts about your chances of getting bowel cancer affected your ability to perform your daily activities?**					

Are you aware that there is a bowel cancer screening programme in England?

- Yes
- No

Do you think that benefits of bowel cancer screening outweigh the possible side effects, potential harms and inconvenience? Please select all that you think apply.

- Yes, for everyone
- No, for everyone
- It depends on your age and sex
- It depends on how you feel about cancer
- It depends on how you feel about screening tests

Have you been sent an invitation to take part in bowel screening in the past?

- Yes
- No
- Don’t know

[The following three questions were only asked to those who say yes to the above question]

If yes, which test(s) were you offered? Tick all that apply

- Stool test to check for blood (FOBt or FIT)
- Sigmoidoscopy
- Colonoscopy

Did you complete the screening test offered?

- Yes
- No

If yes, which tests did you complete and what was the outcome? Tick all that apply

- Stool test to check for blood
  - Normal
  - Abnormal
- Sigmoidoscopy
  - Normal (no polyps)
  - Abnormal (one or more polyps)
- Colonoscopy
  - Normal (no polyps)
  - Abnormal (one or more polyps)

NUMERACY (from Schwartz scale[2])

As the information we will be giving you about bowel cancer screening includes numbers, we would like to know how you answer the following questions.

Imagine we flip a fair coin 1000 times.

**What is your best guess at how many times the coin would come up heads in 1000 flips?**	

In the UK National Lottery®, the chance of winning a £10 prize is 1%.

**What is your best guess at how many people would win a £10 prize if 1000 people each bought a single ticket to UK NATIONAL LOTTERY®?**	

In the EuroMillions® Lottery, the chance of winning a car is 1 in 1000.

**What percent of the EuroMillions® tickets win a car?**	

It is important that you pay attention to this study. Please select "Strongly Disagree"

Strongly disagree Strongly agree

BOWEL CANCER SCREENING

Bowel cancer screening involves having tests to check if you have or are at risk of bowel cancer. Screening can help detect bowel cancer at an early stage, when it is easier to treat. It can also be used to help check for and remove small growths in the bowel called polyps, which can turn into cancer over time. Participation in screening is a personal choice.

There are three main tests used for screening. Over the next few pages of the survey you will see some information about each of these tests. You will then see several comparisons of the potential benefits and harms of screening for one of the screening tests. Please read through the information about the tests carefully so that you can use that information to help you decide whether you would choose to be screened or not

Faecal immunochemical testing (FIT)

Faecal immunochemical testing (FIT) is used to check for tiny amounts of blood in your poo. It doesn't diagnose bowel cancer, but it is a simple way to find out if you need further tests. It should be repeated every other year. You do the test at home by taking a sample of poo and placing it on a special card. You then post the card in a special envelope for analysis. You don’t need to do anything particular before doing the test. There are no complications from the test itself but if a test is positive for blood you will be referred for a colonoscopy (further information provided below). Depending on the findings at that colonoscopy you may then be advised to have additional colonoscopies, typically every three to five years.

Sigmoidoscopy

Sigmoidoscopy is when a thin, flexible tube with a camera at the end is used to look inside your bowel. It is done to look for and remove any small growths called polyps. These could eventually turn into cancer if they're not removed.

The test itself is performed once, at a hospital or clinic. On the day of the test you would need to use an enema about an hour before leaving for your appointment by squeezing the liquid from a clear pouch into your bottom – it will make you poo very soon after you've used it. You are awake during the test and it only takes a few minutes. It is usually painless, although some people find it uncomfortable. If you do have any pain, it should only last a few moments. You can then go home soon after the test is finished. You don't need to stay in hospital overnight. Most people can return to their normal activities the same day.

Sigmoidoscopy screening is usually safe but in rare cases it can cause harm to the bowel. About 1 person in every 3,000 may have serious bleeding caused by bowel scope screening. Sometimes the bowel can be torn during bowel scope screening -- this is even rarer.

If a polyp is seen, it will be removed and you will be referred for a colonoscopy. Depending on the findings at that colonoscopy you may then be advised to have additional colonoscopies, typically every three to five years.

Colonoscopy

Colonoscopy is also when a thin, flexible tube with a camera at the end is used to look inside the bowel to look for and remove any small growths called polyps. It is similar to a sigmoidoscopy except that it checks further up your bowel and takes a longer time.

As with a sigmoidoscopy, it is performed once, at a hospital or clinic. Several days before your colonoscopy you would need to avoid some foods. The day before your colonoscopy you would then need to take a medicine (a laxative) to clear out your bowel. The laxative acts very quickly and will give you diarrhoea. You will probably need to poo several times and will want to stay near a toilet.

At the time of the colonoscopy, you would be offered a sedative. This is usually given as an injection into a vein in your arm. It is to make the colonoscopy more comfortable and to make you more relaxed. With a sedative, most people experience only mild or no pain. It takes between 30 and 45 minutes. After the colonoscopy you will probably feel like resting, so you may want to book the whole day away from your other commitments.

A colonoscopy is usually safe but in rare cases it can cause harm to the bowel. About 1 person in every 400 has bleeding after their colonoscopy but it is usually easy to stop. Rarely, the bleeding is more difficult to stop and means that the person needs to be admitted to hospital. This happens to about 1 in every 2000 people having a colonoscopy. Even more rarely, colonoscopy can cause a small tear in the bowel. This happens to about 1 in every 2500 people having a colonoscopy. Rarely, the combination of the laxative and the sedation can also cause heart or circulatory problems such as low blood pressure, fainting, an irregular heart beat or chest pain.

Depending on the results of the colonoscopy you may be advised to have additional colonoscopies, typically every three to five years.

Based on the information you have just read, how much inconvenience, burden or worry do you associate with having a *faecal immunochemical test* (FIT)?

No inconvenience burden or worry — Very great inconvenience, burden or worry

How much inconvenience, burden or worry do you associate with having a *sigmoidoscopy*?

No inconvenience burden or worry — Very great inconvenience, burden or worry

How much inconvenience, burden or worry do you associate with having a *colonoscopy*?

No inconvenience burden or worry — Very great inconvenience, burden or worry

BOWEL CANCER SCREENING SCENARIOS (including SURE test[4])

In this last section of the survey you will see different scenarios showing comparisons of the number of people who develop bowel cancer, the number of people who die from bowel

cancer and the number of side effects for people who have screening compared to those who do not, all over a 15 year period.

Each of the scenarios will show the outcomes for 1000 people. The difference between the scenarios is the estimated risk at the beginning of those people developing bowel cancer over the 15 year period. People can be at higher or lower risk of developing bowel cancer depending on their age, sex, family history, height and weight, and lifestyle choices, such as the amount of red meat they eat.

After each scenario there will be questions for you to answer at the bottom of the page. Some of the differences between the scenarios are quite subtle so please look carefully at all the text and numbers before answering the questions.

[Only two risk levels are shown below for illustration.]

The picture below shows what would happen over a 15 year period. It compares screening with FIT every two years with no screening in 1000 people with a #% risk of developing bowel cancer.

[Representation of benefits and harms of FIT at #% absolute risk, for example]

Based on the information above, if 1000 people opted for FIT how many cases of bowel cancer would be prevented?

X	X	X	X

Based on this information, would you choose to go for screening?
- Yes
- No

Do you feel sure about the best choice for you?
- Yes
- No

Do you know the benefits and risks of each option?
- Yes
- No

Are you clear about which benefits and risks matter most to you?
- Yes
- No

Do you have enough support and advice from this information and the information provided earlier to make a choice?
- Yes
- No

The picture below shows what would happen over a 15 year period. It compares screening with FIT every two years with no screening in 1000 people with a #% risk of developing bowel cancer.

[Representation of benefits and harms of FIT at #% absolute risk, for example]

Based on this information, would you choose to go for screening?

- Yes
- No

Thank you very much for completing this survey. Your time and contribution is very much appreciated.

References

1 Lerman C, Trock B, Rimer BK, *et al.* Psychological and behavioral implications of abnormal mammograms. *Ann Intern Med* 1991;**114**:657–61.

2 Schwartz LM, Woloshin S, Black WC, *et al.* The role of numeracy in understanding the benefit of screening mammography. *Ann Intern Med* 1997;**127**:966–72. doi:10.7326/0003-4819-127-11-199712010-00003

3 Crockett RA, Weinman J, Hankins M, *et al.* Time orientation and health-related behaviour: Measurement in general population samples. *Psychol Heal* 2009;**24**:333–50. doi:10.1080/08870440701813030

4 Wild K V., Mattek N, Austin D, *et al.* “Are You Sure?” *J Appl Gerontol* 2016;**35**:627–41. doi:10.1177/0733464815570667
