# Supplementary material for: The impact of information about different absolute benefits and harms on intention to participate in colorectal cancer screening: A think-aloud study and online randomised experiment
Source: PLoS One. 2021 Feb 16;16(2):e0246991. doi: 10.1371/journal.pone.0246991 (PMC7886213; doi:10.1371/journal.pone.0246991)
Supplement: S2 File — (PDF) [file pone.0246991.s002.pdf]

Think Aloud study discussion script

This script will act as a guide during the discussion

Welcome

Confirm understanding of the study, participation, recording, confidentiality issues and consent.

Introduction
"The purpose of the study is to find out about your views on bowel cancer screening, the diagnostic tests available to detect bowel cancer and the associated benefits and harms of each.
As we go through the online survey please share your thoughts and understanding of the information provided. I will prompt you during the discussion".

Warm up question
To start the discussion and act as an ice breaker can you tell me *"what did you do today before coming to the session?"*

PROMPT: Encourage the participant to describe and give more information on their activities, meals and snacks eaten, and how they travelled to the session.

Online survey- initial thoughts on background information

"I will now show you the online survey. It will first ask you some questions about yourself such as sex, age and education level. Next, it will give you some information on bowel cancer frequency and then each of the tests available for screening with possible side effects for each. Please discuss your thoughts on this information as you are reading."

The participant will click through each page until they reach the end of this section.

PROMPT: Prompt participant if no views are expressed on reading the summary

"Next the survey will ask you to tell us what you think about the burden and seriousness of the risks associated with each screening test, and with developing bowel cancer."

PROMPT: Prompt participant while they are working through the information.
Potential questions *"Why did you choose that? What are you thinking at this moment?"*

Online survey- risk cases

"Next the survey will give you several examples in which you will be shown images displaying a risk level for developing bowel cancer and the associated risks and benefits associated with choosing to have each screening test. You will be asked to tell whether you would opt for screening based on the information provided in each case".

The participant will complete each of the examples and will be encouraged to describe their thoughts on the graphics displayed and their choices to the following questions.
PROMPT: Prompt participants while they are reading and working out what the information means. Potential questions could include *"What are you thinking while you are looking at this image?" "Can you describe your reasons for choosing this answer".*

Closing questions

Talk through with the participants their experience of looking at each of the examples and their reasons for choosing their answers, encouraging them to share overall views on the possible risk presentation and description of each test.

Ask questions such as *“Would you change any of your answers now that you have completed all of the cases?” “Have your views changed now?” “Do you have any other thoughts?”*

Is there any other information you would have liked to have seen?

Thank participant for taking part, confirm recording and confidentiality.
