# Supplementary material for: The impact of information about different absolute benefits and harms on intention to participate in colorectal cancer screening: A think-aloud study and online randomised experiment
Source: PLoS One. 2021 Feb 16;16(2):e0246991. doi: 10.1371/journal.pone.0246991 (PMC7886213; doi:10.1371/journal.pone.0246991)
Supplement: S1 Table — (PDF) [file pone.0246991.s003.pdf]

S1 Table. Estimates of absolute benefits and risks of screening presented within the scenarios

	Biennial FIT			Sigmoidoscopy			Colonoscopy		
15-year risk of developing CRC	1%	3%	5%	1%	3%	5%	1%	3%	5%
CRC without screening	10	30	50	10	30	50	10	30	50
CRC with screening	9	29	48	7	22	36	7	20	33
Reduction in CRC with screening	1	1	2	3	8	14	3	10	17
Deaths from CRC without screening	3	9	16	3	9	16	3	9	16
Deaths from CRC after screening	2	5	8	1	5	8	1	4	6
Reduction in deaths from CRC with screening	1	4	8	2	4	8	2	5	10
Number needing colonoscopy	160	246	328	82	237	382	1000	1000	1000
Additional colonoscopies	39	121	209	36	103	195	42	124	226
Complications*	1	3	5	1	3	6	1	5	8

* Requiring a visit to the emergency department or hospitalisation
