# Supplementary material for: The impact of information about different absolute benefits and harms on intention to participate in colorectal cancer screening: A think-aloud study and online randomised experiment
Source: PLoS One. 2021 Feb 16;16(2):e0246991. doi: 10.1371/journal.pone.0246991 (PMC7886213; doi:10.1371/journal.pone.0246991)
Supplement: S2 Table — (PDF) [file pone.0246991.s004.pdf]

S2 Table. Characteristics of participants for online survey

		FIT			Sigmoidoscopy			Colonoscopy			Incomplete responses
	n	1—5—3 *n* = 111	3—1—5 *n* = 109	5—3—1 *n* = 107	1—5—3 *n* = 109	3—1—5 *n* = 108	5—3—1 *n* = 108	1—5—3 *n* = 108	3—1—5 *n* = 109	5—3—1 *n* = 109	*n* = 22
Age	978										
45-49		32 (29)	32 (29)	31 (29)	29 (27)	31 (29)	42 (39)	31 (29)	36 (33)	24 (22)	7 (32)
50-54		30 (27)	33 (30)	33 (31)	36 (33)	34 (31)	25 (23)	32 (30)	27 (25)	34 (31)	6 (27)
55-59		23 (21)	22 (20)	17 (16)	20 (18)	21 (19)	17 (16)	18 (17)	24 (22)	24 (22)	5 (23)
60-64		17 (15)	12 (11)	18 (17)	13 (12)	11 (10)	14 (13)	18 (17)	11 (10)	14 (13)	1 (5)
65-69		7 (6)	6 (6)	5 (5)	5 (5)	5 (5)	8 (7)	6 (6)	6 (6)	9 (8)	2 (9)
>70		2 (2)	4 (4)	3 (3)	6 (6)	6 (6)	2 (2)	3 (3)	5 (4)	4 (4)	1 (5)
Sex	978										
Female (n, %)		71 (64)	77 (71)	71 (66)	73 (67)	72 (67)	63 (58)	65 (60)	77 (71)	75 (69)	17 (77)
Ethnicity	972										
White (n, %)		101 (91)	107 (98)	100 (94)	108 (99)	104 (97)	101 (94)	104 (97)	105 (97)	105 (97)	21 (95)
Education	978										
University level (n, %)		62 (56)	50 (46)	48 (45)	60 (55)	53 (49)	50 (46)	46 (43)	53 (49)	49 (45)	9 (41)
Family history of bowel cancer	958	12 (11)	13 (12)	10 (10)	11 (10)	13 (13)	5 (5)	6 (6)	8 (7)	8 (8)	3 (14)
Numeracy	975										
High numeracy (n, %)		85 (77)	81 (74)	93 (87)	87 (80)	88 (81)	81 (75)	84 (79)	86 (80)	83 (77)	16 (73)
Aware of current screening programme	978	82 (74)	74 (68)	77 (72)	84 (77)	80 (74)	81 (75)	78 (72)	79 (72)	74 (68)	17 (77)
History of IBD	968	1 (1)	3 (3)	3 (3)	3 (3)	4 (4)	7 (7)	3 (3)	3 (3)	4 (4)	0 (0)
Prior invitation to screening	978	34 (31)	32 (29)	33 (31)	33 (30)	32 (30)	34 (31)	31 (29)	34 (31)	33 (30)	8 (36)
Prior attendance at screening	978	24 (22)	19 (17)	27 (25)	25 (23)	24 (22)	25 (23)	23 (21)	26 (24)	24 (22)	7 (32)
FIT or FOBt		20 (18)	14 (13)	23 (21)	22 (20)	18 (17)	19 (18)	19 (18)	21 (19)	16 (15)	5 (23)
Sigmoidoscopy		2 (2)	2 (2)	1 (1)	0 (0)	2 (2)	0 (0)	0 (0)	1 (1)	0 (0)	0 (0)
Colonoscopy		2 (2)	3 (3)	3 (3)	3 (3)	4 (4)	6 (6)	4 (4)	4 (4)	8 (7)	2 (9)
Cancer worry (mean, SD)*	966	5.6 (2.2)	5.3 (2.2)	5.8 (2.3)	5.6 (2.2)	5.4 (2.1)	5.3 (1.9)	5.0 (2.0)	5.1 (1.9)	5.4 (2.2)	5.6 (2.2)
Perception of risk of bowel cancer (mean, SD)**	978	3.8 (2.3)	3.4 (2.2)	3.8 (2.2)	3.5 (2.2)	3.7 (2.2)	3.5 (2.5)	3.5 (2.2)	3.2 (2.3)	3.5 (2.3)	3.8 (2.4)
Inconvenience, worry or burden of tests (mean, SD)***											
FIT	978	1.4 (0.7)	1.5 (0.7)	1.5 (0.6)	1.5 (0.7)	1.5 (0.8)	1.4 (0.6)	1.3 (0.5)	1.5 (0.8)	1.6 (0.9)	1.6 (0.7)
Sigmoidoscopy	978	2.5 (1.0)	2.7 (0.9)	2.7 (0.8)	2.7 (0.8)	2.8 (0.9)	2.6 (0.9)	2.3 (0.7)	2.6 (0.9)	2.8 (0.9)	2.5 (0.7)
Colonoscopy	978	3.1 (1.1)	3.3 (1.0)	3.3 (0.9)	3.3 (0.9)	3.6 (1.1)	3.1 (1.1)	3.0 (0.9)	3.1 (1.1)	3.4 (1.0)	3.1 (0.9)

* On a scale of 3-15 ** On a continuous scale from Unlikely (0) to Likely (10) *** On a scale from 1 (No inconvenience, worry or burden) to 5 (Very great inconvenience, worry or burden).
