# Supplementary material for: The impact of information about different absolute benefits and harms on intention to participate in colorectal cancer screening: A think-aloud study and online randomised experiment
Source: PLoS One. 2021 Feb 16;16(2):e0246991. doi: 10.1371/journal.pone.0246991 (PMC7886213; doi:10.1371/journal.pone.0246991)
Supplement: S3 Table — (PDF) [file pone.0246991.s005.pdf]

S3 Table. Intention to attend screening within each study group across all three scenarios

**Group**	**FIT**			**Sigmoidoscopy**			**Colonoscopy**		
**Order**	**1—5—3**	**3—1—5**	**5—3—1**	**1—5—3**	**3—1—5**	**5—3—1**	**1—5—3**	**3—1—5**	**5—3—1**
	n=111	n=109	n=107	n=109	n=108	n=108	n=108	n=109	n=109
Scenario 1 (n, % yes)	86 (77)	75 (69)	83 (78)	86 (79)	69 (64)	81 (75)	75 (69)	75 (69)	81 (74)
Scenario 2 (n, % yes)	99 (89)	63 (58)	66 (62)	90 (83)	59 (55)	74 (69)	83 (77)	54 (50)	74 (68)
Scenario 3 (n, % yes)	91 (82)	78 (72)	56 (52)	85 (78)	77 (71)	58 (54)	80 (74)	83 (76)	53 (49)
