# Supplementary material for: The impact of information about different absolute benefits and harms on intention to participate in colorectal cancer screening: A think-aloud study and online randomised experiment
Source: PLoS One. 2021 Feb 16;16(2):e0246991. doi: 10.1371/journal.pone.0246991 (PMC7886213; doi:10.1371/journal.pone.0246991)
Supplement: S4 Table — (PDF) [file pone.0246991.s006.pdf]

S4 Table. Adjusted percentages ± 95% CI* for participants intending to attend screening at each baseline 15 year percentage risk of CRC and for each order in which participants were presented with the three scenarios.

15-year risk (%)	Order in which participants were presented with the risk levels		
	1—5—3	3—1—5	5—3—1
1	75.3 (70.6-80.0)	54.0 (48.6-59.4)	51.5 (46.1-57.0)
3	78.0 (73.6-82.5)	67.2 (62.1-72.3)	66.0 (60.9-71.2)
5	82.6 (78.5-86.7)	73.0 (68.2-77.9)	75.6 (70.9-80.3)

* These “adjusted” percentages are predicted from multivariable logistic regression model including the screening test and previous invitation to screening in addition to the risk level and order in which the scenarios were presented. They were estimated using the margins command in Stata and are the percentage of responders expected for each outcome, stratified by each category / group, should they have the same levels of all the other covariates in the model as all included survey responders (these percentages are also known as recycled predictions).
