# Supplementary material for: The impact of information about different absolute benefits and harms on intention to participate in colorectal cancer screening: A think-aloud study and online randomised experiment
Source: PLoS One. 2021 Feb 16;16(2):e0246991. doi: 10.1371/journal.pone.0246991 (PMC7886213; doi:10.1371/journal.pone.0246991)
Supplement: S5 Table — (PDF) [file pone.0246991.s007.pdf]

**S5 Table. Odds ratios (ORs) of Intending to attend screening at each baseline percentage risk and for each order in which participants were presented with the three scenarios for participants who got the test question correct or incorrect.** ORs are adjusted for screening test, previous invitation to screening and an interaction between baseline percentage risk and order.

| 15-year risk (%) | Order in which participants were presented with the risk levels | | |
|---|---|---|---|
| | 1—5—3 | 3—1—5 | 5—3—1 |
| **Participants who got the test question correct** | | | |
| 1 | 1 (ref) | 1 (ref) | 1 (ref) |
| 3 | 1.24 (0.95 to 1.62) | 1.93 (1.44 to 2.60) | 2.29 (1.65 to 3.18) |
| 5 | 1.58 (1.18 to 2.12) | 2.67 (1.83 to 3.89) | 3.25 (2.26 to 4.67) |
| **Participants who got the test question incorrect** | | | |
| 1 | 1 (ref) | 1 (ref) | 1 (ref) |
| 3 | 1.05 (0.75 to 1.47) | 1.62 (1.28 to 2.05) | 1.49 (1.16 to 1.92) |
| 5 | 1.53 (0.96 to 2.45) | 2.08 (1.54 to 2.81) | 2.65 (1.89 to 3.74) |
