# Supplementary material for: The impact of information about different absolute benefits and harms on intention to participate in colorectal cancer screening: A think-aloud study and online randomised experiment
Source: PLoS One. 2021 Feb 16;16(2):e0246991. doi: 10.1371/journal.pone.0246991 (PMC7886213; doi:10.1371/journal.pone.0246991)
Supplement: S6 Table — (PDF) [file pone.0246991.s008.pdf]

S6 Table. Adjusted percentages ± 95% CI* for response patterns to the scenarios.

Variable	Always no (%)	Scenario dependent (%)	Always yes (%)	*p* value
Screening test				0.085
FIT	15.7 (12.0-19.4)	29.2 (24.4-34.0)	55.1 (50.1-60.1)	
Sigmoidoscopy	20.5 (16.4-24.5)	21.9 (17.4-26.4)	57.7 (52.7-62.7)	
Colonoscopy	19.2 (15.2-23.2)	29.6 (24.6-34.5)	51.2 (46.1-56.3)	
Age (years)				**0.002**
45-54	15.2 (12.5-17.8)	26.9 (23.2-30.6)	58.0 (54.1-61.9)	
55-64	22.1 (17.6-26.7)	26.9 (21.8-32.0)	51.0 (45.6-56.3)	
>65	35.3 (23.7-47.0)	23.1 (13.0-33.2)	41.6 (30.9-52.2)	
Sex				0.063
Female	19.7 (16.9-22.6)	28.1 (24.7-31.5)	52.2 (48.6-55.8)	
Male	15.9 (12.2-19.7)	24.6 (20.1-29.2)	59.4 (54.5-64.4)	
Ethnicity				**0.046**
White	18.6 (16.3-20.9)	26.2 (23.4-29.0)	55.2 (52.2-58.1)	
Non-white	15.0 (4.7-25.3)	45.3 (29.3-61.3)	39.8 (24.5-55.0)	
University level education				0.92
Yes	18.6 (15.3-21.9)	27.3 (23.5-31.2)	54.1 (50.0-58.1)	
No	18.3 (15.1-21.6)	26.4 (22.3-30.4)	55.3(51.0-59.6)	
Family history				**0.013**
No	18.4 (16.1-20.7)	28.2 (25.3-31.1)	53.4 (50.3-56.4)	
Yes	19.9 (11.3-28.5)	13.5 (6.1-20.8)	66.7 (57.2-76.1)	
Prior history of screening				**0.0002**
No	21.1 (18.2-24.0)	28.0 (24.7-31.3)	50.9 (47.4-54.5)	
Yes	9.3 (5.0-13.6)	23.0 (16.3-29.7)	67.7 (60.5-74.9)	
Numeracy				0.051
Low	19.5 (14.5)	20.0 (14.4-25.6)	60.5 (54.1-66.9)	
High	18.1 (15.5-20.7)	28.8 (25.6-31.9)	53.1 (49.8-56.4)	
Understanding of information				0.58
Incorrect	18.8 (15.5-22.1)	25.4 (21.5-29.3)	55.8 (51.7-60.0)	
Correct	18.1 (14.9-21.3)	28.3 (24.4-32.3)	53.5 (49.4-57.6)	
Prior perceptions of screening				**<0.0001**
Yes for all	8.1 (5.8-10.4)	27.1 (23.6-30.7)	64.8 (61.0-68.6)	
No for all	18.4 (1.5-35.3)	21.2 (3.4-38.9)	60.4 (40.0-81.2)	
It depends	34.7 (29.7-39.7)	27.7 (23.1-32.4)	37.6 (32.5-42.6)	

* These "adjusted" percentages are predicted from multivariable multinomial logistic regression models including all factors in the table in addition to the order in which the scenarios were presented. They are presented here alongside the p-values which are included in the main text in Table 2. They were estimated using the margins command in Stata and are the percentage of responders expected for each outcome, stratified by each category / group, should they have the same levels of all the other covariates in the model as all included survey responders (these percentages are also known as recycled predictions).
